# Long-Term Growth and Neurodevelopmental Outcomes in Children with Cerebral Palsy: A Nationwide Population-Based Study

**DOI:** 10.3390/children13070917

**Published:** 2026-07-11

**Authors:** Sung-Hoon Chung, Young Hwa Song, Jae Woo Lim

**Affiliations:** 1 Department of Pediatrics, Kyung Hee University College of Medicine, Kyung Hee University Hospital at Gangdong, Seoul 05278, Republic of Korea; 2 Department of Pediatrics, Konyang University College of Medicine, Daejeon 35365, Republic of Korea; 3 Myunggok Medical Research Center, Konyang University College of Medicine, Daejeon 35365, Republic of Korea; 4 Department of Pediatrics, Konyang University Hospital, 158 Kwanjeodong-ro, Seo-gu, Daejeon 35365, Republic of Korea

**Keywords:** cerebral palsy, neonatal outcomes, neurocognitive development, growth disorders, cohort studies

## Abstract

**Highlights:**

**What are the main findings?**

- Growth failure and neurodevelopmental impairments in children with cerebral palsy progressively worsen and the gap compared to peers widens between 30–36 months and 54–60 months.
- In the overall birth cohort, hypoxic–ischemic encephalopathy and necrotizing enterocolitis were associated with poor long-term growth and development independently of cerebral palsy.

**What are the implications of the main findings?**

- The widening relative disparity in developmental delay between children with and without CP during the preschool transition emphasizes the critical need for sustained, long-term monitoring beyond early infancy.
- Targeted multidisciplinary nutritional interventions are essential, particularly for high-risk subgroups with HIE or NEC, to mitigate the widening developmental and growth gaps.

**Abstract:**

**Background/Objectives:** Although cerebral palsy (CP) is a leading cause of childhood disability, population-based data on long-term growth trajectories and domain-specific developmental outcomes remain limited. This study aimed to investigate the prevalence of growth failure and neurodevelopmental delay, along with the associated perinatal risk factors, in Korean children with CP. **Methods:** We conducted a nationwide, retrospective cohort study using data from the National Health Insurance Service. A birth cohort of 1,256,934 infants born between 2013 and 2015 was linked to claims and infant health screening data, followed through 2020. Longitudinal outcomes were assessed at 30–36 months and 54–60 months. Multivariable logistic regression identified risk factors for growth failure (height/weight/head circumference < 3rd percentile) and developmental delay (K-DST score < −2 SD). **Results:** The overall prevalence of CP was 0.18%. Outcomes for affected children progressively worsened over time. Specifically, the relative disparity in developmental delay between children with and without CP widened over time, with the adjusted odds ratio increasing from 30–36 to 54–60 months. In the multivariable analysis, CP diagnosis showed the strongest independent association with adverse outcomes. Regarding neonatal morbidities, hypoxic–ischemic encephalopathy (HIE) and necrotizing enterocolitis (NEC) were associated with the most pronounced and persistent deficits across the overall cohort, independently of CP. The association between bronchopulmonary dysplasia and developmental delay was attenuated and no longer significant at 54–60 months, whereas its association with growth failure persisted. **Conclusions:** Children with CP face profound and widening deficits in somatic growth and neurodevelopment throughout early childhood. Persistent growth failure, particularly in patients with HIE or NEC, underscores the critical need for early multidisciplinary nutritional intervention to mitigate long-term adverse outcomes.

## 1. Introduction

Cerebral palsy (CP) is one of the conditions most often associated with persistent motor problems in children. Reports from several high-income countries usually place the prevalence somewhere between 1.5 and 3.5 per 1000 live births, although the exact numbers differ by region and study method. Children diagnosed with CP do not only have motor impairments [1,2]. Many also experience difficulties with growth, delays in different areas of development, and various medical issues that tend to persist over time and influence both daily life and healthcare use [3]. While a recent nationwide study confirmed the declining incidence of CP in Korea [4], detailed population-based data on long-term growth trajectories and domain-specific developmental milestones stratified by neonatal risk factors remain limited.

The strongest risk factors for CP identified in past large-scale population-based studies are premature birth and low birth weight (BW), with extreme premature birth conferring a substantially higher risk than term infants [4,5]. Perinatal brain injuries, such as intraventricular hemorrhage (IVH) and periventricular leukomalacia (PVL), and neonatal complications, including respiratory distress syndrome (RDS) and necrotizing enterocolitis (NEC), have been associated with increased odds of developing CP in nationwide cohort analyses [6,7]. Previous longitudinal registry and insurance claims studies have further revealed that children with CP experience markedly higher all-cause mortality and that underweight status is a strong independent predictor of adverse outcomes [8]. However, most studies in the literature have focused on short-term outcomes or have used facility-based samples, leaving significant gaps in our understanding of long-term growth trajectories and neurodevelopmental milestones at the population level.

The Korean National Health Insurance Service (NHIS) database, which covers virtually the entire population, offers a unique opportunity to examine these outcomes with minimal selection bias and extended follow-up through early childhood [9]. In this study, we aimed to investigate the prevalence of growth failure and neurodevelopmental delay, as well as the associated perinatal risk factors, among Korean children with CP using linked NHIS claims data from a national birth cohort. By analyzing a nationwide cohort, this study has delineated the risk profiles associated with adverse long-term growth and neurodevelopmental outcomes in children with CP.

## 2. Materials and Methods

### 2.1. Study Population and Data Sources

This population-based retrospective cohort study used data from the Korean NHIS, a nationwide population surveillance system that covers nearly the entire pediatric population in Korea. We constructed a birth cohort by identifying all live births between 1 January 2013, and 31 December 2015. In order to obtain comprehensive health data, we linked health insurance claims tracked through 31 December 2020, to identify clinical diagnoses, including CP and neonatal morbidities, with the infant and child health screening database to evaluate longitudinal growth and developmental outcomes. This linkage allowed for the comprehensive reconstruction of a longitudinal cohort from administrative surveillance data. From the initial birth cohort of 1,256,934 live births, we identified participants who attended specific screening rounds designated for primary and secondary analyses. The final analytical sample consisted of children with valid anthropometric and developmental screening measurements at each time point. For the primary analysis at 30–36 months (4th screening), the analytical sample included 1,002,694 children for growth assessment and 1,002,650 children for developmental screening. For the secondary analysis at 54–60 months (6th screening), the sample consisted of 879,230 and 879,150 children, respectively.

### 2.2. Definition of CP

CP was identified by the presence of ICD-10 diagnostic codes G80–G83 recorded in the National Health Insurance database. There is no predetermined age cutoff for the diagnosis of CP. Children were classified as having CP if they had any CP-related ICD-10 code at any point during the follow-up period until the end of 2020. This approach reflects the reality of insurance claims-based epidemiology, in which diagnoses are documented at the time of clinical contact.

### 2.3. Neonatal Morbidities

Major neonatal complications were identified using ICD-10 diagnostic codes recorded during hospital admission. RDS was coded as P220, P228, or P229. Bronchopulmonary dysplasia (BPD) was identified using the code P27x (any fourth digit encompassing mild, moderate, or severe disease). IVH was coded as P102, P520, P521, P522, or P523, whereas PVL was identified using the code P911 or P912. NEC was coded as P77x (any fourth digit), and hypoxic–ischemic encephalopathy (HIE) was identified using the codes P910 or P916. Patent ductus arteriosus (PDA) was coded as Q250, and persistent pulmonary hypertension of the newborn (PPHN) was coded as P293. Retinopathy of prematurity (ROP) was coded as H351, and neonatal seizures were identified using the code P90x (any fourth digit). These diagnoses were recorded during inpatient admission, primarily during the NICU stay. According to standard claims-based coding practices, new morbidity codes for these conditions were unlikely to be appended during subsequent non-NICU hospitalizations.

### 2.4. Study Design and Outcomes

The primary outcome was the presence of a CP diagnosis in claims records. Secondary outcomes included associations between the neonatal morbidities and CP occurrence and long-term growth and neurodevelopmental outcomes assessed at two key time points: 30–36 months and 54–60 months of age, which correspond to the 4th and 6th rounds of the Korean National Health Screening Program for infants and children and key transitional periods for preschool integration. Accordingly, CP was analyzed in two distinct roles: as the outcome in the etiological analysis of neonatal morbidities, and, separately, as an independent exposure variable—alongside BW, sex, and neonatal morbidities—in the prognostic analyses of growth and developmental outcomes. These are conceptually distinct models addressing different questions, rather than a single model in which CP is simultaneously outcome and predictor. For growth analysis, BW was categorized into four groups: <1000 g, 1000–1499 g, 1500–2499 g, and ≥2500 g (reference category). Growth failure was defined as height, weight, or head circumference below the 3rd percentile using the Korean National Growth Standards [10]. For the developmental outcomes, the Korean Developmental Screening Test (K-DST) was used to assess six domains: gross motor function, fine motor function, cognition, language, social function, and self-care. Developmental delay was operationally defined as a screening-positive status, as indicated by a K-DST score below −2 standard deviations requiring further evaluation [11]. Both male and female infants were included in all of the analyses. Sex information was obtained from NHIS eligibility data. BW was selected as the primary exposure because it reflects early physical status and is strongly associated with perinatal complications and adverse outcomes. Gestational age (GA) was not entered as a candidate variable in the models, and collinearity between GA and BW was not formally assessed; a GA-adjusted BW metric was not used. The implications of omitting GA are addressed in the Limitations.

### 2.5. Statistical Analysis

For the categorical variables, including the prevalence of neonatal morbidities and neurodevelopmental outcomes, comparisons between the CP and non-CP groups were performed using the chi-squared test. We fitted multivariable logistic regression models to identify the independent factors associated with growth failure and developmental delay at 30–36 and 54–60 months. For growth failure, separate models were used for height, weight, and head circumference. In all models, the ≥2500 g BW group was used as the reference category. Independent variables included BW, sex, and major neonatal morbidities (BPD, IVH, NEC, and HIE). PVL was excluded from the multivariable models because of its exceptionally strong bivariate association with CP (CP prevalence 28.61% among infants with PVL, the highest of any morbidity examined, exceeding even that of HIE at 20.88%). aORs with 95% confidence intervals (CI) and *p* values are reported. Statistical significance was set at *p* < 0.05. All of the statistical analyses were performed using SAS version 9.4 (SAS Institute, Cary, NC, USA). All analyses at each screening stage were conducted as available case analyses, without imputation. Given the very large sample size, statistical significance was readily attained even for small absolute differences; interpretation therefore emphasizes the magnitude of aORs and absolute differences rather than *p* value thresholds alone. *p* values were not adjusted for multiple comparisons, and borderline associations should be interpreted accordingly.

## 3. Results

### 3.1. Study Population and Prevalence of CP

Among 1,256,934 children born between 2013 and 2015, 2245 cases of CP were identified, corresponding to an overall prevalence of 0.18% (Table 1). The cohort comprised 645,147 males (51.3%) and 611,787 females (48.7%) with a male-to-female CP prevalence ratio of 0.20% to 0.16% (*p* < 0.001). The prevalence of CP showed marked variation across GA categories. Children born at extremely early GAs (≤27 weeks) had the highest prevalence at 10.75%, followed by those born at 28–31 weeks (5.3%), 32–36 weeks (0.79%), and ≥37 weeks (0.11%). Similarly, the prevalence of CP decreased substantially with increasing BW. Infants weighing less than 1000 g had a prevalence of 9.72%, compared to 5.29% for those weighing 1000–1499 g, 0.78% for those weighing 1500–2499 g, and 0.12% for those weighing 2500 g or more. Temporal trends in CP prevalence across the three birth cohort years (2013–2015) demonstrated relative stability within each GA and BW stratum (Supplementary Table S1). Among the infants born at ≤27 weeks of gestation, CP prevalence declined from 12.07% in 2013 to 8.19% in 2015 (*p* = 0.006). For those born at ≥37 weeks of age, the prevalence decreased from 0.12% to 0.10% (*p* = 0.008). The BW-stratified trends were similar, with the lowest BW group showing a decline from 10.44% in 2013 to 7.46% in 2015 (*p* = 0.021), and the highest BW category showing a decrease from 0.12% to 0.10% (*p* = 0.019). These patterns reflected the overall decline in the prevalence of CP observed in the Korean population during this period.

### 3.2. Association Between Neonatal Morbidities and CP

All major neonatal complications were significantly associated with an increased risk of CP (Table 2). PVL showed the highest association, with a CP prevalence of 28.61%, compared to 0.15% without PVL. HIE demonstrated a CP prevalence of 20.88% versus 0.17% without HIE. IVH, neonatal seizures, BPD, and ROP were associated with CP prevalences of 8.07%, 9.20%, 9.94%, and 6.64%, respectively, compared with 0.15–0.17% in unaffected infants. RDS, PDA, PPHN, and NEC showed CP prevalences of 5.73%, 4.87%, 6.45%, and 5.91%, respectively (*p* < 0.001 for all).

### 3.3. Growth Failure and Developmental Outcomes at 30–36 and 54–60 Months

**Figure 1:**
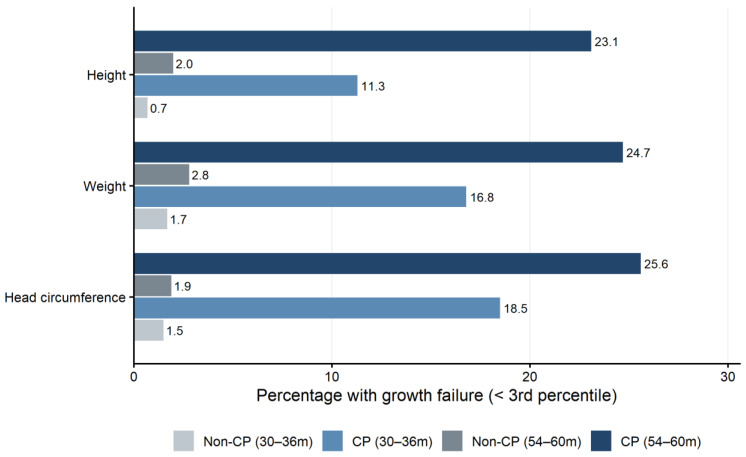
Comparison of the growth failure rates between children with and without CP at 30–36 months (4th screening) and 54–60 months (6th screening). The bar charts show the percentage of children with height, weight, and head circumference below the 3rd percentile. The children with CP exhibited significantly higher growth failure rates than the non-CP group across all measures and time points. All comparisons were considered statistically significant (*p* < 0.001). CP = cerebral palsy.

Children with CP demonstrated substantially higher rates of growth failure than their peers without CP across all anthropometric measures at both assessment time points. At 30–36 months, 11.3% of children with CP had heights below the 3rd percentile compared to 0.7% of children without CP, 16.8% versus 1.7% for weight, and 18.5% versus 1.5% for head circumference. These disparities persisted and widened by 54–60 months, with 23.1% of children with CP showing height growth failure (2.0% in the non-CP group), 24.7% versus 2.8% for weight, and 25.6% versus 1.9% for head circumference. The magnitude of the difference between the CP and non-CP groups remained consistent across both time points, with children with CP experiencing 11–16-fold higher rates of growth failure (Figure 1).

**Figure 2:**
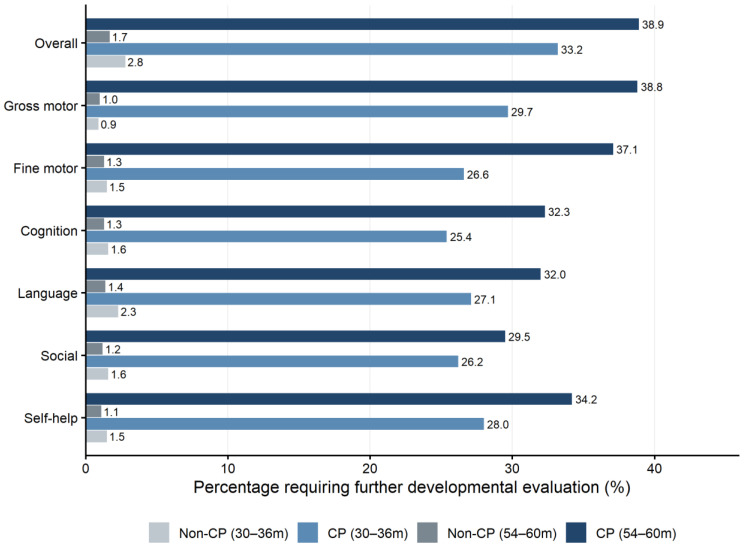
Comparison of developmental delay rates across six domains in children with and without CP at 30–36 months (4th screening) and 54–60 months (6th screening). The bar charts illustrate the percentage of children requiring further developmental evaluation (K-DST score < −2 SD). Children with CP showed significantly higher rates of delay in all domains than those in the non-CP group. Gross motor delay was most prevalent in the CP group, and the gap between the groups widened over time. All comparisons were considered statistically significant (*p* < 0.001). K-DST = Korean Developmental Screening Test; CP = cerebral palsy.

Developmental assessments using the K-DST revealed similarly pronounced disparities between groups across all six developmental domains. At 30–36 months, 33.2% of children with CP required further developmental evaluation (overall K-DST score < −2 SD) compared to 2.8% of non-CP children. By 54–60 months, this gap widened further, with 38.9% of children with CP versus 1.7% of children without CP requiring further evaluation. When examined by individual domains, gross motor delay was most prevalent in children with CP at both time points (29.7% at 30–36 months and 38.8% at 54–60 months), followed by fine motor delays (26.6% and 37.1%, respectively). Self-help, language, and cognitive delays ranged from 25.4% to 29.7% at 30–36 months and 29.5% to 34.2% at 54–60 months, whereas social delays were relatively lower at both time points (26.2% and 29.5%, respectively) (Figure 2). In contrast, children without CP showed consistently low rates of delay across all domains, remaining below 3% at both assessment points.

### 3.4. Multivariable Analysis of Factors Associated with Growth Failure

At 30–36 months, the multivariable logistic regression analysis demonstrated that CP diagnosis was independently associated with substantially elevated odds of growth failure across all three anthropometric measures (Table 3). The children with CP had aOR of 5.75 (95% CI 4.55–7.27) for height growth failure, 4.06 (95% CI 3.33–4.95) for weight, and 6.00 (95% CI 4.96–7.25) for head circumference growth failure (*p* < 0.001 for all). BW showed strong dose-dependent associations with growth failure: infants weighing less than 1000 g had aOR of 31.67 for height (95% CI 24.88–40.31), 41.56 for weight (95% CI 34.66–49.84), and 28.94 for head circumference (95% CI 23.65–35.41) compared to those ≥2500 g. The intermediate BW categories demonstrated proportionally lower aOR, with 1000–1499 g showing aOR of 7.61–12.78 and 1500–2499 g showing aOR of 3.43–4.70 across the three measures. Male sex was associated with higher odds of weight growth failure (aOR 1.17, 95% CI 1.13–1.20) but lower odds of head circumference growth failure (aOR 0.87, 95% CI 0.85–0.90). Among the neonatal complications, HIE showed the strongest association with growth failure across all three anthropometric measures (aOR 2.41–3.54, all *p* < 0.001), followed by NEC (aOR 1.38–1.54, all *p* ≤ 0.008). BPD and IVH showed modest associations with weight and head circumference failure but were not significantly associated with height growth failure.

Similar patterns persisted at 54–60 months (Table 4), with CP remaining significantly associated with growth failure across all measures (aOR 4.60–6.83, all *p* < 0.001). BW associations remained pronounced, with less than 1000 g conferring aOR of 13.81–21.74 compared to ≥2500 g. HIE maintained the strongest association among neonatal complications (aOR 2.32–4.60, all *p* < 0.001), while NEC associations decreased in magnitude but remained significant for both height and head circumference. IVH showed a significant association with head circumference failure at this later time point (aOR 1.37, 95% CI 1.15–1.63, *p* < 0.001).

### 3.5. Multivariable Analysis of Factors Associated with Developmental Delay

At 30–36 months, the multivariable logistic regression analysis revealed that CP diagnosis was independently associated with substantially elevated odds of developmental delay (aOR 10.69, 95% CI 9.25–12.37, *p* < 0.001) (Table 5). BW showed dose-dependent associations, with less than 1000 g conferring aOR of 3.53 (95% CI 2.83–4.41) compared to ≥2500 g. Male sex was associated with higher odds of developmental delay (aOR 2.81, 95% CI 2.73–2.89). Among the neonatal complications, HIE showed the strongest association (aOR 2.36, 95% CI 1.73–3.23, *p* < 0.001), followed by NEC and BPD (aOR 1.50 and 1.36, respectively). At 54–60 months, CP diagnosis showed a markedly stronger association with developmental delay relative to children without CP, with the aOR nearly doubling to 20.42 (95% CI 17.73–23.53, *p* < 0.001) (Table 6), indicating a widening disparity over time. BW associations persisted with similar dose-dependent patterns, whereas male sex maintained an elevated risk (aOR 2.04, 95% CI 1.97–2.11). HIE and NEC remained the strongest associated neonatal complications (aOR 1.90 and 1.58, respectively), though BPD and IVH associations diminished and did not reach statistical significance at this later time point.

**Figure 3:**
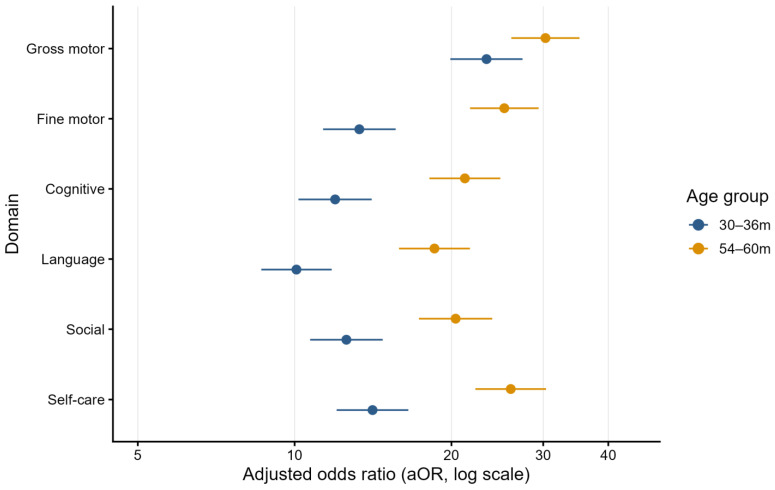
Adjusted odds ratios (aORs) for developmental delay by domain in children with CP. The forest plot displays the aORs with 95% confidence intervals for each of the six developmental domains at 30–36 and 54–60 months of age. Between the two time points, the aOR for delay increased substantially across all domains, with gross motor function showing the strongest association. The blue circles represent the 30–36 months age group, and the yellow circles represent the 54–60 months age group. CP = cerebral palsy.

When stratified by developmental domain, CP showed pronounced associations across all six domains at both time points, with distinctive patterns. At 30–36 months, gross motor delay demonstrated the highest CP association (aOR 23.35, 95% CI 19.90–27.38), followed by self-care (aOR 14.11, 95% CI 12.04–16.53) and fine motor (aOR 13.31, 95% CI 11.34–15.63), while language showed the lowest association (aOR 10.08, 95% CI 8.63–11.78) with cognitive and social domains showing intermediate values. By 54–60 months, the aOR estimates increased substantially across all of the domains, with gross motor reaching 30.3 (95% CI 26.06–35.22), self-care 25.99 (95% CI 22.23–30.38), and fine motor 25.26 (95% CI 21.71–29.40), while language remained relatively lower at 18.55 (95% CI 15.86–21.70) (Figure 3). The consistent pattern across both time points highlights the motor domains as particularly vulnerable to CP, with language preservation representing relative strength.

## 4. Discussion

In this nationwide cohort, we examined the long-term growth and neurodevelopmental outcomes in children with CP across the entire Korean population. We found that deficits in children with CP progressively worsened from 30–36 to 54–60 months, with the disparity in developmental delay relative to peers widening over this interval (aOR 10.69 at 30–36 months vs. 20.42 at 54–60 months). CP diagnosis showed the strongest independent association with adverse outcomes across all of the domains tested, even after adjusting for BW and neonatal morbidities. Beyond motor impairment, CP-affected children showed substantial delays across all developmental domains, including gross motor, fine motor, self-care, cognition, language, and social development, indicating multidomain neurodevelopmental involvement. Head circumference growth failure was found to be most strongly associated with CP, suggesting that microcephaly serves as a robust marker of severe perinatal brain injury and an adverse developmental trajectory. Our observed period prevalence of 1.8 per 1000 is broadly consistent with the current pooled birth prevalence of 1.6 per 1000 (95% CI 1.5–1.7) reported for high-income countries, and remains well below the markedly higher estimates from low- and middle-income countries (birth prevalence up to 3.4 per 1000, and period prevalence up to 3.7 per 1000) [2]. As period prevalence is generally higher than birth prevalence, our estimate sitting slightly above the high-income birth-prevalence figure is expected and does not indicate a genuinely elevated occurrence. The comparison is nonetheless limited by our claims-based ascertainment, which credits a CP diagnosis from a single coded clinical contact, whereas clinical registries such as Surveillance of Cerebral Palsy in Europe and the Australian Cerebral Palsy Register confirm persistent motor impairment at 4–5 years of age and exclude children whose signs later resolve [2]; near-universal NHIS coverage may also reduce under-ascertainment relative to registries that rely on voluntary reporting.

Children with CP in this cohort demonstrated progressive worsening of growth failure from 30–36 months to 54–60 months across all anthropometric measures, with the growth gap between children with CP and non-CP children widening substantially over time rather than narrowing. Longitudinal registry studies consistently document that children with CP have lower mean height, weight, and body mass index z-scores than reference populations and that growth deficits accumulate progressively with age, particularly in those with more severe motor impairment (higher Gross Motor Function Classification System levels) [12,13,14]. This progressive divergence reflects the complex interplay between nutritional and non-nutritional mechanisms. Nutritional contributors include oral motor dysfunction, dysphagia, gastroesophageal reflux disease, and prolonged mealtimes, which collectively limit energy intake and increase feeding difficulties for caregivers [15,16]. Non-nutritional mechanisms include abnormal muscle tone, reduced physical activity, chronic respiratory complications, and endocrine disturbances such as alterations in the growth hormone axis, which operate independently of dietary intake to constrain linear growth [17,18,19].

Head circumference growth failure warrants particular attention, as it was the anthropometric measure most strongly associated with CP in our cohort (aOR 6.0–6.83 across time points). Microcephaly is a common finding in CP, and it serves as a clinical marker of a more extensive early brain injury rather than a purely nutritional problem [20]. Population-based CP registries have demonstrated that reduced head circumference is strongly associated with severe intellectual disability, epilepsy, and higher motor functional impairment levels, thus suggesting that head growth failure reflects impaired brain growth and neural development [21,22]. Moreover, past studies of children with developmental disabilities have shown that microcephaly is associated with lower cognitive test scores and poorer adaptive functioning across multiple domains, including communication and daily living skills [21,23]. These findings support the interpretation that persistent head-growth failure in our cohort is not solely attributable to inadequate nutrition but rather reflects the underlying severity of perinatal brain injury, and that microcephaly may serve as a robust clinical predictor of adverse long-term neurodevelopmental outcomes. Although both optimized nutritional support and multidisciplinary growth surveillance can improve weight gain in some children with CP, linear growth and head circumference often remain subnormal, indicating that structural brain damage and systemic factors fundamentally limit the growth potential in this population [24,25].

In this nationwide cohort, the aOR for developmental delay (defined as a K-DST total score < −2 SD) in children with CP relative to those without almost doubled between 30–36 and 54–60 months of age, indicating that affected children progressively fall further behind their peers rather than showing catch-up over time; the crude proportion requiring further evaluation rose more modestly, from 33.2% to 38.9%. This pattern is consistent with longitudinal studies of children with CP showing that early profiles of motor, language, and communication functions are strongly predictive of later outcomes. Many children with more severe motor or communication impairments show only limited catch-up and remain well below age-level expectations across childhood, with the gap in everyday functioning compared to typically developing peers tending to widen over time [26,27]. Our finding that CP remained the strongest determinant of developmental delay after adjusting for BW and neonatal morbidities suggests that underlying brain injury and its functional consequences largely shape long-term developmental trajectories despite the advances seen in neonatal care and early rehabilitation. Domain-specific analyses further showed that gross motor delay was most strongly associated with CP, but substantial impairments were also evident in the fine motor and self-care domains, which is consistent with previous studies showing that greater motor severity and higher GMFCS scores are closely linked to limitations in daily activities and functional independence in children with CP [28,29]. Population-based studies have reported high rates of language and communication impairments among school-aged children with CP, with receptive and expressive trajectories frequently remaining below age expectations, particularly in those with co-occurring cognitive impairment [30,31]. Narrative reviews and cohort studies have also indicated that cognitive difficulties and executive dysfunction contribute to poorer academic achievement and school outcomes, even in the absence of formal intellectual disability, emphasizing the multidomain nature of CP-related developmental burden [32,33].

The worsening developmental trajectory observed in our cohort may reflect a “growing into a deficit” phenomenon, in which children with CP do not fully recover lost neural function despite early intervention, but instead show increasingly complex functional impairments as age-related demands rise [34]. Although early intervention can make use of the critical windows of neuroplasticity, particularly in the first 2–3 years of life, animal and human neuroimaging studies have suggested that bilateral or extensive brain injuries have a limited capacity for compensatory reorganization, which constrains the extent of functional recovery that can be achieved through rehabilitation alone [35]. Consistent with this, randomized trials and follow-up studies have indicated that although early and intensive interventions can improve functional outcomes at school age, such programs rarely eliminate the developmental gap relative to typically developing peers [36,37], which is in line with the persistent and even increasing aOR of delay observed at 54–60 months in this cohort.

Furthermore, the nearly twofold increase in the aOR for developmental delay between 30–36 and 54–60 months in this cohort coincided with a critical developmental transition toward school readiness when demands shifted from basic motor and cognitive skills to more complex social-emotional competence, self-regulation, and academic preparation [38]. Preschool-aged children with CP are known to show substantial difficulties across key readiness domains, including mobility, self-care, social function, and communication, with more than half exhibiting delayed communication skills [39]. These observations have highlighted the need not only for intensive early rehabilitation in infancy but also for targeted school-readiness interventions during preschool years to support social integration and academic success at school entry.

Although CP diagnosis itself showed the strongest association with adverse outcomes, HIE and NEC were the neonatal morbidities most strongly associated with growth failure and developmental delay across the cohort, independently of CP. We did not test these associations specifically within the CP subgroup; nonetheless, the biological mechanisms are well described. Unlike the focal white matter injuries often seen in preterm-born CP (e.g., PVL), HIE frequently involves global or multifocal damage to deep gray matter structures [40], which is closely linked to persistent oropharyngeal dysfunction and swallowing difficulties that limit caloric intake and may drive severe growth failure [41]. Similarly, NEC was independently associated with poorer growth and developmental outcomes across the cohort. This is consistent with the “gut–brain axis” hypothesis, in which intestinal injury triggers a systemic inflammatory response; the released pro-inflammatory cytokines can cross the blood–brain barrier and exacerbate white matter injury, thereby impairing long-term neurodevelopment [42,43]. Furthermore, in infants requiring surgical intervention for NEC, chronic malabsorption and short bowel syndrome act as additional biological constraints on long-term growth [44]. In contrast, the association between BPD and developmental delay was attenuated and no longer statistically significant at 54–60 months (aOR 1.23, *p* = 0.079), whereas its association with growth failure persisted. We interpret this attenuation cautiously: it may reflect genuine change, but could equally arise from selective attendance at later screening, survivor bias, unmeasured BPD severity, the absence of gestational-age adjustment, or reduced statistical power, and it should not be taken as evidence of a specific physiological “pulmonary catch-up” [45]. The persistence of growth failure likely reflects the chronic metabolic burden of the disease: children with BPD often exhibit increased resting energy expenditure due to greater work of breathing, so that caloric resources are preferentially allocated to respiratory maintenance rather than somatic growth [46].

In children with CP, the progressive widening of the growth gap observed in this study underscores the critical need for early and aggressive nutritional intervention. This necessity is particularly acute for those with a history of HIE or NEC, who exhibit the most severe deficits. Passive monitoring is insufficient; previous longitudinal studies have demonstrated that timely interventions, such as the introduction of high-calorie enteral formulas or percutaneous endoscopic gastrostomy, can significantly improve weight gain and stabilization of growth velocity in children with moderate-to-severe CP [47]. However, nutritional management alone should not be the sole intervention. A multidisciplinary approach involving pediatric gastroenterologists, neurologists, and rehabilitation specialists is required. Recent evidence indicates that the implementation of a specialized Pediatric Nutritional Support Team (NST) significantly improves nutritional status and caregiver satisfaction when compared to standard care [48]. Therefore, for the high-risk CP subgroups identified in our study, specifically those with structural brain injury or intestinal sequelae, clinicians should consider early referral to specialized nutritional teams in order to bridge the developmental gap before it becomes irreversible [49].

This study has several strengths. First, it utilized a large nationally representative dataset from the Korean NHIS, minimizing the selection bias often found in single-center or hospital-based studies. Second, by linking claims data with serial K-DST results, we evaluated long-term functional outcomes, specifically, growth trajectories and developmental domains, rather than relying solely on diagnostic codes. This longitudinal approach allowed us to capture the progressive divergence in development that might have been missed in cross-sectional analyses. Given the retrospective, observational design, however, all findings should be interpreted as associations rather than causal effects.

This study also has several limitations. First, CP was identified from any ICD-10 code G80–G83 recorded during follow-up, without repeated coding, an age threshold, or clinical confirmation. As only G80 is specific to CP—G81–G83 may also capture non-CP paralytic syndromes—this may reduce specificity and over-include cases. A G80-only definition with G80–G83 as a sensitivity analysis would be preferable but could not be performed, as data access has expired. Second, CP-coded children were not cross-referenced with genetic or syndromic diagnoses, so some children with monogenic or chromosomal conditions that mimic or coexist with CP may be included. Third, BW was derived from ICD-10 code bands rather than a measured value, which may misclassify infants near band boundaries; a continuous or gestational-age-adjusted metric could not be constructed. Fourth, GA was never entered into the models and its collinearity with BW was not formally tested, so residual confounding cannot be excluded, and the BW associations should be read as reflecting prematurity and growth restriction jointly rather than BW independent of GA. Fifth, neither CP-stratified models nor CP-by-morbidity interaction models were fitted, so whether HIE or NEC effects differ specifically within children with CP could not be tested. Sixth, analyses used available cases only, without multiple imputation or inverse-probability weighting, and included-versus-excluded comparisons could not be constructed after data access ended. Because attrition was substantial (20.2% by 30–36 months, 30.1% by 54–60 months) and children with CP had far higher mortality than those without (5.9% vs. 0.23%), the most severely affected children were likely underrepresented at follow-up, biasing our estimates toward the null. The disparities reported here are therefore likely conservative. Seventh, outcomes were modelled separately at each round with cross-sectional logistic regression, which does not account for within-subject correlation between the two assessments or model individual trajectories; a generalized estimating equations or mixed-effects approach on continuous z-scores would be preferable and is a priority for future work. Eighth, the K-DST is a parent-reported screening tool, and the <−2 SD cutoff is a referral threshold rather than a diagnosis, so dichotomization may not capture the full severity gradient. Finally, *p* values were not adjusted for multiple comparisons, and borderline associations should be interpreted with caution. Several of these limitations reflect the intrinsic constraints of administrative claims data and the time-limited nature of NHIS data access, and each represents a specific direction for future studies with continued data access.

## 5. Conclusions

This nationwide study confirms that children with CP face profound and progressively widening deficits in both somatic growth and neurodevelopment. The relative disparity in developmental delay compared to typically developing peers widens between 30–36 and 54–60 months (aOR 10.69 vs. 20.42), illustrating a distinct “growing into a deficit” pattern during the preschool transition. Outcomes are associated with neonatal history: in the overall cohort, HIE and NEC showed the strongest associations with severe and persistent impairments, independently of CP. In contrast, the association between BPD and developmental delay attenuated over time while its association with growth failure persisted. To mitigate these widening long-term gaps, a paradigm shift toward early multidisciplinary nutritional support and targeted school-readiness interventions is essential, particularly for the most vulnerable subgroups identified in this study.

## Figures and Tables

**Table 1:** Baseline characteristics of the study population (2013–2015).

Characteristic	Total (*n*)	CP Cases (*n*)	CP Prevalence (%)	*p*
Sex				
Male	645,147	1279	0.20	<0.001
Female	611,787	966	0.16	
Gestational age (weeks)				
≤27	2550	274	10.75	<0.001
28–31	6148	326	5.30	
32–36	37,022	294	0.79	
≥37	1,211,214	1351	0.11	
Birth weight (g)				
<1000	2429	236	9.72	<0.001
1000–1499	4615	244	5.29	
1500–2499	49,238	383	0.78	
≥2500	1,200,652	1382	0.12	
Total	1,256,934	2245	0.18	

Data presented as number (*n*) and percentage. *p* values calculated using chi-square tests for each subgroup category. CP = cerebral palsy.

**Table 2:** Association between neonatal morbidities and cerebral palsy.

Variable	With Morbidity		Without Morbidity		*p*
	Total *N*	CP Cases, *n* (%)	Total *N*	CP Cases, *n* (%)	
RDS	11,345	650 (5.73)	1,245,589	1595 (0.13)	<0.001
BPD	3248	323 (9.94)	1,253,686	1922 (0.15)	<0.001
PDA	10,018	488 (4.87)	1,246,916	1757 (0.14)	<0.001
PPHN	1659	107 (6.45)	1,255,275	2138 (0.17)	<0.001
IVH	2727	220 (8.07)	1,254,207	2025 (0.16)	<0.001
PVL	1220	349 (28.61)	1,255,714	1896 (0.15)	<0.001
NEC	1421	84 (5.91)	1,255,513	2161 (0.17)	<0.001
ROP	6475	430 (6.64)	1,250,459	1815 (0.15)	<0.001
HIE	819	171 (20.88)	1,256,115	2074 (0.17)	<0.001
Neonatal seizure	2871	264 (9.20)	1,254,063	1981 (0.16)	<0.001

Values are presented as *n* (%). *n*, number of CP cases; *N*, total number of infants in each group. *p* values were calculated using chi-square tests. CP = cerebral palsy, RDS = respiratory distress syndrome, BPD = bronchopulmonary dysplasia, PDA = patent ductus arteriosus, PPHN = persistent pulmonary hypertension of the newborn, IVH = intraventricular hemorrhage, PVL = periventricular leukomalacia, NEC = necrotizing enterocolitis, ROP = retinopathy of prematurity, HIE = hypoxic–ischemic encephalopathy.

**Table 3:** Factors associated with growth failure (<3rd percentile) at 30–36 months of age: a multivariable logistic regression analysis.

Variable	Adjusted Odds Ratio (95% Confidence Interval)					
	Height	*p*	Weight	*p*	Head Circumference	*p*
CP	5.75 (4.55–7.27)	<0.001	4.06 (3.33–4.95)	<0.001	6.00 (4.96–7.25)	<0.001
Birth weight (g)						
<1000	31.67 (24.88–40.31)	<0.001	41.56 (34.66–49.84)	<0.001	28.94 (23.65–35.41)	<0.001
1000–1499	11.69 (9.81–13.93)	<0.001	12.78 (11.28–14.47)	<0.001	7.61 (6.56–8.84)	<0.001
1500–2499	3.86 (3.60–4.15)	<0.001	4.70 (4.49–4.91)	<0.001	3.43 (3.26–3.62)	<0.001
≥2500 (ref)	1.0	-	1.0	-	1.0	-
Sex						
Male	1.05 (1.00–1.10)	0.031	1.17 (1.13–1.20)	<0.001	0.87 (0.85–0.90)	<0.001
Female (ref)	1.0	-	1.0	-	1.0	-
BPD	0.98 (0.79–1.22)	0.870	1.22 (1.04–1.45)	0.017	1.26 (1.05–1.52)	0.015
IVH	0.96 (0.75–1.23)	0.749	1.04 (0.87–1.25)	0.632	1.20 (0.99–1.45)	0.069
NEC	1.54 (1.17–2.03)	<0.001	1.41 (1.13–1.74)	<0.001	1.38 (1.09–1.76)	0.008
HIE	2.41 (1.47–3.94)	<0.001	2.44 (1.69–3.52)	<0.001	3.54 (2.51–4.99)	<0.001

Data are presented as adjusted odds ratios (95% confidence intervals). *p* values < 0.05 are considered statistically significant. Ref indicates the reference group. CP = cerebral palsy, BPD = bronchopulmonary dysplasia, IVH = intraventricular hemorrhage, NEC = necrotizing enterocolitis, HIE = hypoxic–ischemic encephalopathy.

**Table 4:** Factors associated with growth failure (<3rd percentile) at 54–60 months of age: a multivariable logistic regression analysis.

Variable	Adjusted Odds Ratio (95% Confidence Interval)					
	Height	*p*	Weight	*p*	Head Circumference	*p*
CP	6.43 (5.44–7.59)	<0.001	4.60 (3.90–5.42)	<0.001	6.83 (5.78–8.07)	<0.001
Birth weight (g)						
<1000	13.81 (11.28–16.89)	<0.001	21.74 (18.32–25.81)	<0.001	21.56 (17.81–26.10)	<0.001
1000–1499	5.98 (5.18–6.90)	<0.001	8.12 (7.22–9.14)	<0.001	6.31 (5.46–7.29)	<0.001
1500–2499	2.98 (2.84–3.13)	<0.001	3.70 (3.55–3.85)	<0.001	3.41 (3.25–3.58)	<0.001
≥2500 (ref)	1.0	-	1.0	-	1.0	-
Sex						
Male	0.95 (0.92–0.98)	0.001	1.16 (1.13–1.19)	<0.001	1.08 (1.05–1.12)	<0.001
Female (ref)	1.0	-	1.0	-	1.0	-
BPD	0.99 (0.82–1.19)	0.897	1.21 (1.03–1.41)	0.017	1.28 (1.07–1.53)	0.006
IVH	0.95 (0.78–1.16)	0.625	0.93 (0.79–1.11)	0.423	1.37 (1.15–1.63)	<0.001
NEC	1.42 (1.13–1.80)	0.003	1.14 (0.92–1.41)	0.226	1.31 (1.04–1.65)	0.022
HIE	2.99 (2.18–4.11)	<0.001	2.32 (1.70–3.17)	<0.001	4.60 (3.46–6.12)	<0.001

Data are presented as adjusted odds ratios (95% confidence intervals). *p* values < 0.05 are considered statistically significant. Ref indicates the reference group. CP = cerebral palsy, BPD = bronchopulmonary dysplasia, IVH = intraventricular hemorrhage, NEC = necrotizing enterocolitis, HIE = hypoxic–ischemic encephalopathy.

**Table 5:** Multivariable regression analysis for developmental delay (K-DST total score < −2 SD, requiring further evaluation) at 30–36 months of age: adjusted odds ratios (95% CI).

Variable	Adjusted Odds Ratio (95% CI)	*p*
CP	10.69 (9.25–12.37)	<0.001
Birth weight (g)		
<1000	3.53 (2.83–4.41)	<0.001
1000–1499	2.27 (1.95–2.66)	<0.001
1500–2499	1.56 (1.48–1.64)	<0.001
≥2500 (ref)	1.0	
Sex		
Male	2.81 (2.73–2.89)	<0.001
Female (ref)	1.0	
BPD	1.36 (1.13–1.65)	0.001
IVH	0.99 (0.81–1.21)	0.937
NEC	1.50 (1.18–1.90)	0.001
HIE	2.36 (1.73–3.23)	<0.001

Data are presented as adjusted odds ratios (95% confidence intervals). *p* values < 0.05 are considered statistically significant. Ref indicates the reference group. CP = cerebral palsy, BPD = bronchopulmonary dysplasia, IVH = intraventricular hemorrhage, NEC = necrotizing enterocolitis, HIE = hypoxic–ischemic encephalopathy.

**Table 6:** Multivariable regression analysis for developmental delay (K-DST total score < −2 SD, requiring further evaluation) at 54–60 months of age: adjusted odds ratios (95% CI).

Variable	Adjusted Odds Ratio (95% CI)	*p*
CP	20.42 (17.73–23.53)	<0.001
Birth weight (g)		
<1000	3.77 (2.90–4.89)	<0.001
1000–1499	2.07 (1.70–2.52)	<0.001
1500–2499	1.70 (1.59–1.81)	<0.001
≥2500 (ref)	1.0	
Sex		
Male	2.04 (1.97–2.11)	<0.001
Female (ref)	1.0	
BPD	1.23 (0.98–1.54)	0.079
IVH	1.22 (0.97–1.52)	0.083
NEC	1.58 (1.19–2.10)	0.002
HIE	1.90 (1.32–2.73)	0.001

Data are presented as adjusted odds ratios (95% confidence intervals). *p* values < 0.05 are considered statistically significant. Ref indicates the reference group. CP = cerebral palsy, BPD = bronchopulmonary dysplasia, IVH = intraventricular hemorrhage, NEC = necrotizing enterocolitis, HIE = hypoxic–ischemic encephalopathy.

## Data Availability

The data used in this study were provided by the National Health Insurance Service (NHIS) of Korea (NHIS-2021-4-007). Restrictions apply to the availability of these data, which were used under license for the current study and are not publicly available. Data are, however, available from the NHIS (https://nhiss.nhis.or.kr) with permission from the Institutional Review Board of Korea.

## Supplementary Materials

The following supporting information can be downloaded at: https://www.mdpi.com/article/10.3390/children13070917/s1, Table S1. Prevalence of cerebral palsy according to birth year, gestational age, birth weight (2013–2015).

## Abbreviations

The following abbreviations are used in this manuscript:

aOR	Adjusted Odds Ratio
BPD	Bronchopulmonary Dysplasia
BW	Birth Weight
CI	Confidence Interval
CP	Cerebral Palsy
GA	Gestational Age
HIE	Hypoxic–Ischemic Encephalopathy
IVH	Intraventricular Hemorrhage
K-DST	Korean Developmental Screening Test
NEC	Necrotizing Enterocolitis
NHIS	National Health Insurance Service
PDA	Patent Ductus Arteriosus
PPHN	Persistent Pulmonary Hypertension of the Newborn
PVL	Periventricular Leukomalacia
ROP	Retinopathy of Prematurity
SD	Standard Deviation
