# Supplementary material for: Long-Term Growth and Neurodevelopmental Outcomes in Children with Cerebral Palsy: A Nationwide Population-Based Study
Source: Children (Basel). 2026 Jul 11;13(7):917. doi: 10.3390/children13070917 (PMC13406830; doi:10.3390/children13070917)
Supplement: Supplementary file 1 [file children-13-00917-s001.zip › children-4368545-supplementary.pdf]

Supplementary Table S1. Prevalence of cerebral palsy according to birth year, gestational age, birth weight (2013–2015).

Category	Subgroup	2013	2014	2015	Total	*p*
Gestational age (weeks)	≤ 27	12.07 (95/787)	12.39 (102/823)	8.19 (77/940)	10.75 (274/2,550)	0.006
	28–31	5.23 (111/2,124)	5.26 (104/1,976)	5.42 (111/2,048)	5.30 (326/6,148)	0.957
	32–36	0.92 (105/11,418)	0.79 (95/12,017)	0.69 (94/13,587)	0.79 (294/37,022)	0.129
	≥ 37	0.12 (497/408,169)	0.11 (448/393,408)	0.10 (406/409,637)	0.11 (1,351/1,211,214)	0.008
Birth weight (g)	< 1,000	10.44 (83/795)	11.33 (91/803)	7.46 (62/831)	9.72 (236/2,429)	0.021
	1,000–1,499	5.85 (88/1,505)	5.47 (80/1,463)	4.61 (76/1,647)	5.29 (244/4,615)	0.283
	1,500–2,499	0.86 (134/15,594)	0.74 (120/16,321)	0.74 (129/17,323)	0.78 (383/49,238)	0.373
	≥ 2,500	0.12 (503/404,604)	0.12 (458/389,637)	0.10 (421/406,411)	0.12 (1,382/1,200,652)	0.019

Values are presented as percentages (number of cerebral palsy cases / total births). *P* values were calculated using chi-square tests across birth years within each subgroup.
